# Design of Fe_2_TiO_5_-PDA Heterojunction for Photocatalytic CO_2_ Reduction: From Mechanism Research to Virtual–Real Hybrid Chemistry Experimental Teaching Reform

**DOI:** 10.3390/molecules31101703

**Published:** 2026-05-18

**Authors:** Kai Wang, Yihui Du, Liang Wang

**Affiliations:** 1 College of Urban and Environmental Sciences, Hubei Normal University, Huangshi 435002, China; 17683908397@163.com; 2 School of Chemistry, Chemical Engineering and Biotechnology, Nanyang Technological University, Singapore 637459, Singapore; 3 Institute of Nanochemistry and Nanobiology, School of Environmental and Chemical Engineering, Shanghai University, Shanghai 200444, China

**Keywords:** Fe_2_TiO_5_, polydopamine, heterojunction, photocatalytic CO_2_ reduction, virtual–real hybrid, experimental teaching

## Abstract

Photocatalytic reduction of CO_2_ to produce high-value chemical fuels is a research hotspot for sustainable development, yet its integration into undergraduate experimental teaching is hindered by a high risk, high cost, and shortage of large-scale instruments. Herein, a Fe_2_TiO_5_–polydopamine (PDA) S-scheme heterojunction photocatalyst was fabricated via in situ self-polymerization, and its structure, photoelectric properties, and CO_2_ reduction mechanism were systematically characterized. Under visible light, the heterojunction delivers a CO production rate of 14.1 μmol·g^−1^·h^−1^ (6.6 times that of pure Fe_2_TiO_5_) with 94.2% cyclic stability. More importantly, this work constructs a virtual–real hybrid experimental teaching mode (virtual simulation pre-training + offline practical verification) for inorganic and environmental chemistry experiments, developing a virtual simulation platform with six modules (laboratory safety, instrument introduction, experimental principle, 3D simulation, virtual assessment, and after-school thinking). This mode solves the teaching bottlenecks of high-risk operation and inaccessible large-scale characterization (in situ XPS and CO_2_-BET), standardizes experimental operations, and deepens students’ understanding of photocatalytic mechanisms. This study not only provides a high-efficiency photocatalyst for CO_2_ reduction but also offers a replicable virtual–real integration paradigm for inorganic chemistry experimental teaching reform.

## 1. Introduction

The relentless consumption of fossil fuels has driven atmospheric CO_2_ concentration beyond the critical threshold of 420 ppm, intensifying the greenhouse effect and precipitating an imminent energy crisis that collectively threatens global sustainable development [1,2,3]. Photocatalytic CO_2_ reduction, which harnesses solar energy to convert CO_2_ into value-added chemical fuels such as CO, CH_4_, and HCOOH, stands out as a pivotal technology to realize the “dual carbon” goals by virtue of its synergistic merits of carbon mitigation and renewable energy generation [4,5,6]. The practical implementation of this technology hinges critically on the rational design and fabrication of photocatalyst systems that are highly efficient, robust, and environmentally benign.

Inorganic chemistry experiment is the foundational course for chemistry, environmental engineering and materials majors, but the photocatalytic CO_2_ reduction experiment involving electrospinning synthesis, expensive large-scale instruments (in situ XPS and GC) and toxic reagents faces difficulties in carrying out in local universities, which is consistent with the teaching pain points of Fe-based nanomaterial electrospinning experiments. Traditional experimental teaching is limited to teacher demonstrations, resulting in low students’ operational ability and poor cognitive depth of catalytic mechanisms. Therefore, the key challenge is not simply how to show students an advanced photocatalytic experiment, but how to redesign the experiment as a teachable research process in which safety awareness, standard operation, data literacy, and mechanism-oriented thinking can be trained step by step.

A fundamental challenge in photocatalysis is the rapid recombination of photogenerated electron–hole pairs, which severely undermines quantum efficiency [7,8,9,10]. To circumvent this issue, heterojunction engineering has emerged as a powerful strategy [11,12]. Among various architectures, the S-scheme heterojunction has recently garnered significant attention because it uniquely combines spatial charge separation with preservation of the strongest redox potentials [13,14,15,16]. Unlike conventional type-II or Z-scheme configurations, the S-scheme heterojunction exploits an interfacial built-in electric field to drive the recombination of photogenerated carriers possessing lower redox ability, while retaining electrons in the conduction band and holes in the valence band that exhibit the strongest reducing and oxidizing power, respectively [17,18,19]. This distinctive charge transfer mechanism not only suppresses carrier recombination but also maximizes the thermodynamic driving force for CO_2_ reduction, offering a compelling platform for developing high-performance photocatalysts.

Iron titanate (Fe_2_TiO_5_) with an ilmenite-type structure has emerged as a promising visible-light-responsive photocatalyst owing to its narrow bandgap (~2.2 eV), excellent chemical stability, and earth-abundant composition [20,21,22]. Nevertheless, pristine Fe_2_TiO_5_ suffers from fast charge recombination and a paucity of active sites, which limit its photocatalytic CO_2_ reduction efficiency [23,24]. To overcome these drawbacks, constructing a heterojunction with a suitable partner is imperative. Polydopamine (PDA), a bio-inspired conductive polymer rich in catechol and amine functional groups, possesses exceptional metal-chelating ability and electron transport properties [25,26,27,28]. Therefore, the construction of an Fe_2_TiO_5_-PDA S-scheme heterojunction and the systematic elucidation of its “interfacial charge transfer–catalytic activity–reaction mechanism” relationship represent a frontier in photocatalytic CO_2_ reduction research.

Virtual simulation experiment technology, with the advantages of high safety, low cost, repeatability and immersive interaction, has become an effective way to solve the above teaching bottlenecks, conforming to the OBE (Outcome-Based Education) concept of “student-centered” [29,30]. However, existing virtual simulation cases only focus on single instrument operation, lacking the systematic integration of “synthesis—characterization–performance–mechanism” research chain and failing to realize the “virtual for practical use” of inorganic chemistry experimental teaching reform [31]. In the fields of catalytic oxidation, environmental analysis, Fe-based nanomaterial synthesis, and comprehensive photocatalysis, reported teaching cases have also emphasized the advantages of virtual simulation for high-risk, high-cost, and instrument-intensive experimental contents.

Based on the reform idea of “virtual simulation pre-training first, then practical experimental verification” in Fe-based nanomaterial electrospinning experiments, this work takes Fe_2_TiO_5_-PDA S-scheme heterojunction photocatalytic CO_2_ reduction as the carrier and designs a virtual–real hybrid experimental teaching module covering the whole research chain. This module realizes the organic integration of cutting-edge photocatalytic research and inorganic chemistry experimental teaching, providing a new path for the reform of high-risk and high-cost inorganic comprehensive experiments. This module fully covers all aspects of the catalyst, including X-ray diffraction, scanning electron microscopy morphology characterization, photocatalytic CO_2_ reduction performance testing (CO production rate and cycle stability), photophysical properties (DRS and PL), and reaction mechanism exploration (in situ XPS and CO_2_-BET adsorption), and it realizes the systematic correlation of “structure–photophysical behavior–catalytic activity–reaction mechanism” through interactive comparison of the measured data with the standard spectra built into the virtual simulation platform. The above design aims to provide a virtual–real integration path with high safety, low cost, and a strong exploration nature for environmental engineering experimental teaching. Accordingly, the present manuscript strengthens the educational function of each scientific result: XRD/SEM data are used to train structure identification, DRS/PL data are used to train photophysical interpretation, CO_2_-BET and in-situ XPS are used to train mechanism reasoning, and photocatalytic CO_2_ reduction data are used to train evidence-based evaluation of catalytic performance. This design highlights a full-cycle learning route of “problem introduction—virtual exploration—real verification—data diagnosis—mechanism construction—reflective innovation”.

## 2. Results and Discussion

**Figure 1:**
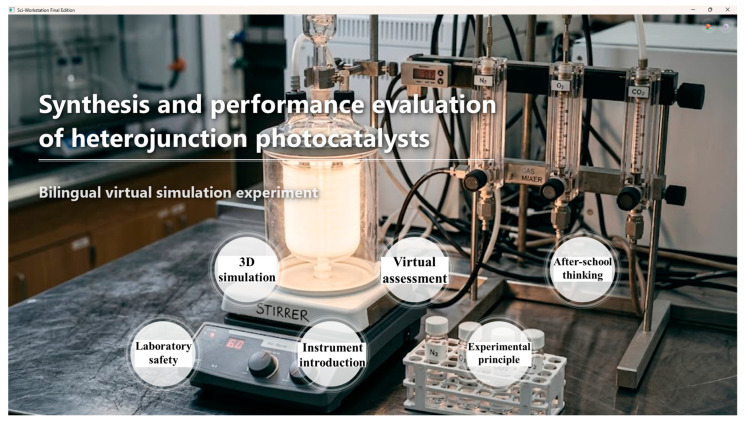
The interface of the virtual simulation software (V1.0).

The virtual simulation platform consists of six modules: laboratory safety, instrument introduction, experimental principles, three-dimensional simulation, virtual testing, and post-class reflection (Figure 1). Students complete the learning of safety regulations, understanding of instrument structure, and comprehension of the heterojunction principle in sequence. Then, they enter the three-dimensional simulation module, where they repeatedly practice key operations such as material weighing, assembly of the reaction vessel (with particular attention to the cleanliness of the inner lining and the installation of the sealing ring), and high-temperature crystallization under real-time system prompts. Finally, they consolidate their learning through virtual testing and post-class reflection. Based on the above simulation results, the students carried out further experimental operations to synthesize the Fe_2_TiO_5_, PDA, and Fe_2_TiO_5_/PDA heterojunction, which was then used for the subsequent analysis and testing. This virtual pre-training method is consistent with the teaching reform idea of Fe-based nanomaterial electrospinning synthesis experiments, realizing “promoting reality through virtualization”. The six modules are organized according to the learning sequence of “knowing the risk—understanding the instrument—explaining the principle—operating the process—diagnosing the data—reflecting on improvement”. In the instrument-introduction module, students rotate and disassemble the virtual electrospinning device, photocatalytic reactor, gas chromatograph, XRD, SEM, CO_2_-BET analyzer, and in-situ XPS system to understand the role of each component. In the experimental-principle module, the S-scheme charge-transfer path, CO_2_ adsorption and activation, and PDA-assisted interfacial electron transfer are visualized through animations. In the 3D simulation module, students repeatedly operate weighing, solution preparation, electrospinning parameter setting, thermal treatment, PDA self-polymerization, photocatalytic reactor sealing, irradiation, gas sampling, and chromatographic analysis. Each key step is linked to a prompt, error warning, or short question, which allows the teacher to use log records as formative evidence of students’ preparation quality. A distinctive feature of this platform is that the virtual experiment is not limited to “watching” or “clicking through” standard procedures. Students are required to identify the cause, revise the operation, and compare the corrected virtual data with real experimental data. This design turns virtual simulation into a diagnostic training environment and prepares students for research-like uncertainty in the real laboratory.

### 2.1. Structure and Morphology

**Figure 2:**
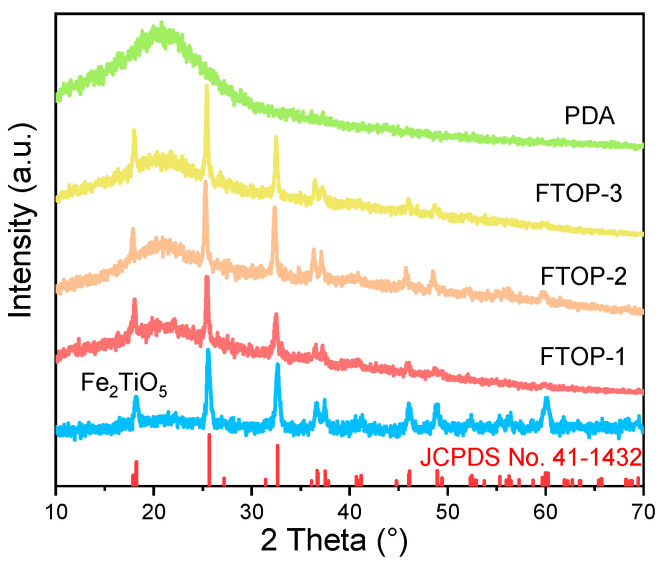
The XRD patterns of the prepared catalyst.

The crystal structure and phase purity of the as-prepared samples were characterized by X-ray diffraction (XRD). As shown in Figure 2, pristine Fe_2_TiO_5_ exhibits characteristic diffraction peaks at 18.2°, 25.4°, 32.5°, 36.5°, and 37.3°, which can be indexed to the (200), (110), (023), (130), and (113) planes, respectively, in good agreement with standard JCPDS card No. 41-1432 for orthorhombic Fe_2_TiO_5_ [32,33]. After the introduction of PDA, no additional diffraction peaks corresponding to PDA are observed in the XRD patterns of the Fe_2_TiO_5_-PDA heterojunctions. This absence suggests that PDA is amorphous or present as a thin layer on the surface of Fe_2_TiO_5_ nanofibers, which does not form a separate crystalline phase. All characteristic peaks of Fe_2_TiO_5_ are retained in the composite samples without any detectable shift in peak positions, indicating that the incorporation of PDA does not alter the crystal structure or lattice parameters of Fe_2_TiO_5_.

**Figure 3:**
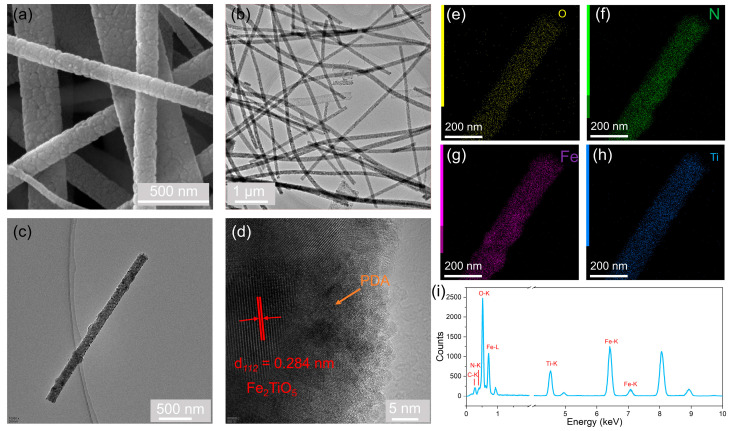
(**a**) SEM image of Fe_2_TiO_5_; (**b**) TEM image of Fe_2_TiO_5_; (**c**,**d**) HRTEM images of the Fe_2_TiO_5_-PDA heterojunction; (**e**–**h**) EDS elemental mapping images of O, N, Fe, and Ti, respectively; (**i**) EDS energy spectrum.

The morphology and microstructure of the as-prepared samples were characterized by SEM and TEM. The SEM (Figure 3a and Figure S1) and TEM (Figure 3b) images of Fe_2_TiO_5_ reveal a clear nanofiber morphology. This one-dimensional fibrous structure is beneficial for photocatalytic applications because it provides a large specific surface area, facilitates light harvesting through multiple scattering, and offers short charge carrier diffusion pathways to the surface. For the Fe_2_TiO_5_-PDA heterojunction, the HRTEM images (Figure 3c,d) reveal an intimate and continuous contact interface between Fe_2_TiO_5_ and the PDA layer, with no visible gap or amorphous barrier. Such intimate contact is crucial for efficient interfacial charge transfer, as it minimizes the recombination loss of photogenerated electron–hole pairs across the heterojunction. The measured lattice spacing in Figure 3d corresponds to the (112) plane of Fe_2_TiO_5_, confirming the crystalline nature of the Fe_2_TiO_5_ component [34,35]. The EDS energy spectrum (Figure 3i and Figure S2) provides semi-quantitative evidence for the coexistence of all constituent elements (Fe, Ti, and O from Fe_2_TiO_5_; C and N from PDA) in the heterojunction material. Through the above analysis, students can intuitively understand the physical form of a “heterogeneous structure”. This sequence helps them move from image observation to structural evidence construction.

### 2.2. Performance Test

**Figure 4:**
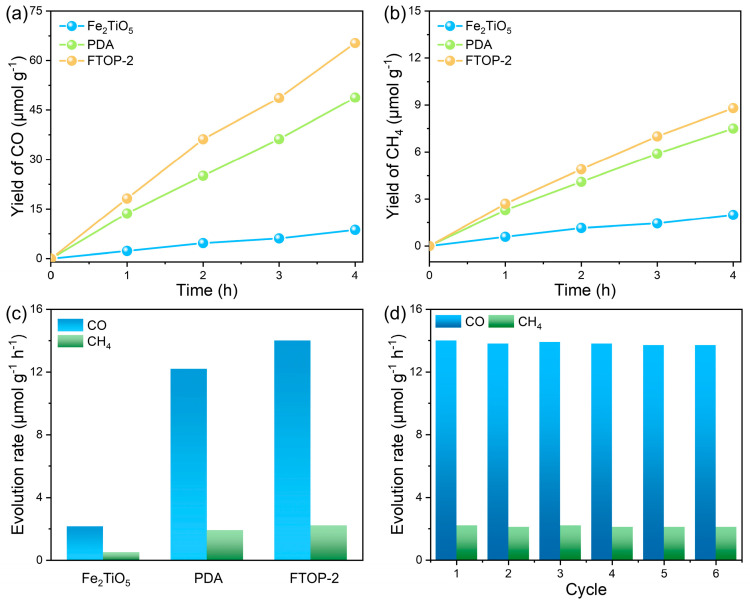
Time-dependent (**a**) CO and (**b**) CH_4_ evolution over Fe_2_TiO_5_-PDA under visible light irradiation; (**c**) comparison of average CO evolution rates over different catalysts; (**d**) recycling stability test of Fe_2_TiO_5_-PDA for 5 consecutive cycles (4 h per cycle).

The photocatalytic CO_2_ reduction activity of the as-prepared samples was evaluated in a gas–solid phase reaction system under visible light irradiation. As shown in Figure 4a,b, the yields of CO and CH_4_ increase linearly with reaction time (0–4 h) without reaching a plateau, indicating that the catalyst maintains stable activity throughout the test period without deactivation. This linear behavior suggests that the surface-active sites are not poisoned or saturated under the experimental conditions. Figure 4c summarizes the average product yields. Pristine Fe_2_TiO_5_ exhibits a low CO yield of only 2.14 μmol·g^−1^·h^−1^, which is attributed to its wide bandgap and rapid charge recombination. In contrast, the Fe_2_TiO_5_-PDA heterojunction achieves a CO yield of 14.1 μmol·g^−1^·h^−1^, approximately 6.6 times higher than that of pure Fe_2_TiO_5_. This significant improvement is attributed to the efficient carrier separation efficiency and enhanced visible light absorption ability provided by the S-scheme heterojunction.

Control experiments conducted under N_2_ atmosphere, under anhydrous conditions, in the absence of catalyst, and under dark conditions yielded no detectable CO or CH_4_, confirming that the products originate exclusively from photocatalytic CO_2_ reduction over the catalyst under light irradiation (Figure S4). To further confirm the carbon source of the products, isotopic labeling experiments were conducted using ^13^CO_2_ as the substrate. GC-MS analysis of the products detected a dominant peak at *m*/*z* = 29, corresponding to ^13^CO, along with a peak at *m*/*z* = 17 attributed to a ^13^CH_4_ fragment (Figure S5). No significant signals were observed at *m*/*z* = 28 or 16, ruling out potential carbon contamination from surface residues, PDA, or organic species, and confirming that the products derive exclusively from photocatalytic CO_2_ reduction. The stability of the catalyst is an important indicator for evaluating its practical value. The cyclic stability test (Figure 4d) showed that after five consecutive cycles (each 4 h), the CO yield of Fe_2_TiO_5_-PDA remained at 94.2% of the initial value, without significant attenuation. A comparison of the photocatalytic CO_2_ reduction performance of FTOP-2 with recently reported catalysts is provided in Table S1. The FTOP-2 heterojunction exhibits a competitive CO production rate relative to other Fe- and TiO_2_-based photocatalysts under similar conditions.

### 2.3. Optical Properties

**Figure 5:**
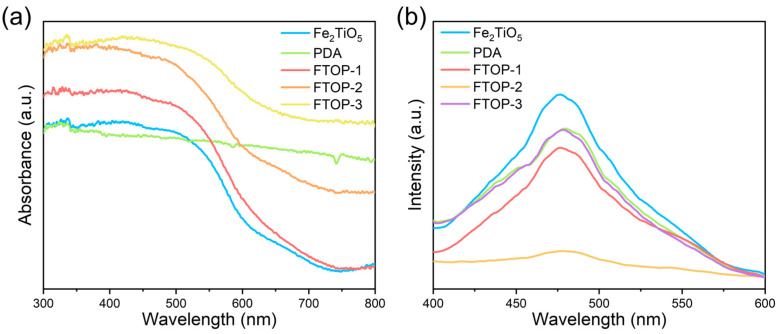
(**a**) DRS spectra and (**b**) PL spectra of prepared catalysts.

The optical properties and charge carrier recombination behavior of the as-prepared samples were investigated by UV–vis diffuse reflectance spectroscopy (DRS) and photoluminescence (PL) spectroscopy. As shown in Figure 5a, pristine Fe_2_TiO_5_ exhibits an absorption edge at approximately 650 nm, corresponding to its intrinsic bandgap. The optical band gap of Fe_2_TiO_5_ was determined to be 1.99 eV using the Tauc equation with (αhν)^2^ versus photon energy (hν) (Figure S6), indicating a direct band gap transition, consistent with previous reports for orthorhombic Fe_2_TiO_5_. PDA alone shows a broad absorption across the entire visible light region. With increasing PDA loading, the Fe_2_TiO_5_-PDA heterojunctions display a progressively enhanced absorbance in the visible range, accompanied by a red shift of the absorption edge [36,37,38]. This observation indicates that PDA acts as a photosensitizer, extending the light-harvesting capability of Fe_2_TiO_5_ into the visible region. By linking spectrum processing with band-gap estimation, the module shifts students from passive spectrum reading to quantitative photophysical analysis.

The photoluminescence (PL) spectra of the samples are presented in Figure 5b. The PL intensity of the Fe_2_TiO_5_-PDA heterojunctions is significantly lower than that of pristine Fe_2_TiO_5_, indicating that the formation of the heterojunction effectively suppresses the recombination of photogenerated electron–hole pairs. Among all the heterojunction samples, FTOP-2 exhibits the lowest PL peak intensity, suggesting that an appropriate amount of PDA (2.0 mg DA·HCl) provides the most efficient interfacial charge separation. This suppressed recombination provides direct evidence for the enhanced photocatalytic activity of the Fe_2_TiO_5_/PDA heterojunction.

### 2.4. Mechanism Exploration

**Figure 6:**
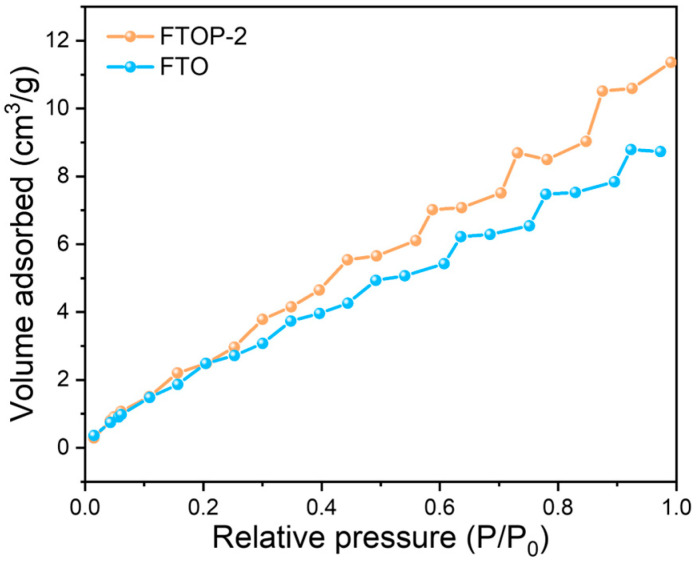
CO_2_ adsorption isotherms of pristine Fe_2_TiO_5_ and Fe_2_TiO_5_-PDA heterojunctions.

The CO_2_ adsorption capacities of the samples were evaluated by CO_2_-BET adsorption measurements. As shown in Figure 6, the Fe_2_TiO_5_-PDA heterojunctions exhibit significantly higher CO_2_ adsorption isotherms compared to pristine Fe_2_TiO_5_. Among them, the FTOP-2 sample achieves a CO_2_ uptake of 11.36 cm^3^ g^−1^. This enhanced adsorption is attributed to the abundant amine functional groups present in PDA, which possess a strong affinity toward weakly acidic CO_2_ molecules [39,40,41]. The improved CO_2_ capture capability directly contributes to the photocatalytic performance, allowing students to establish a clear structure–activity relationship between high adsorption capacity and high catalytic activity.

**Figure 7:**
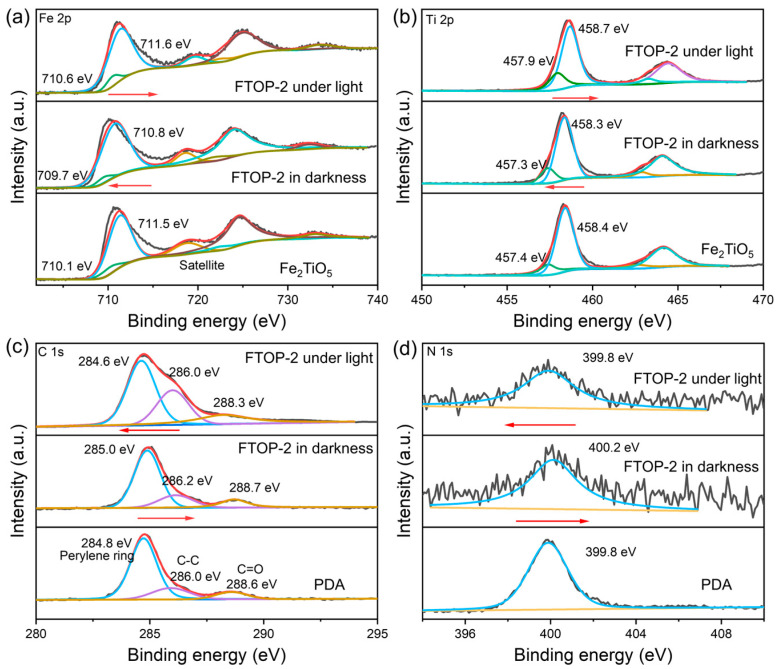
High-resolution XPS spectra of (**a**) Fe 2*p*, (**b**) Ti 2*p*, (**c**) C 1*s* and (**d**) N 1*s* under dark and light illumination.

X-ray photoelectron spectroscopy (XPS) was performed under dark and light illumination to probe the surface electronic states and charge transfer behavior of the Fe_2_TiO_5_-PDA heterojunction (FTOP-2). The quantitative binding energy data for Fe 2*p*, Ti 2*p*, C 1*s*, and N 1*s* are summarized in Table S1. In pristine Fe_2_TiO_5_, the Fe 2*p*_3/2_ and Ti 2*p*_1/2_ peaks are centered at 711.5 eV and 458.4 eV (Figure 7a), respectively, while in pristine PDA, the C 1*s* (Figure 7c) and N 1*s* (Figure 7d) peaks appear at 284.8 eV and 399.8 eV, respectively. In FTOP-2 under dark conditions, Fe 2*p*_3/2_ shifts downward by 0.7 eV to 710.8 eV and Ti 2*p*_1/2_ shifts downward by 0.1 eV to 458.3 eV, indicating an increase in electron density around the Fe and Ti sites due to spontaneous electron transfer from PDA to Fe_2_TiO_5_ upon heterojunction formation. Correspondingly, C 1*s* shifts upward by 0.2 eV to 285.0 eV and N 1*s* shifts upward by 0.4 eV to 400.2 eV, reflecting electron depletion from PDA (Figure 7b). Upon light illumination, Fe 2*p*_3/2_ and Ti 2*p*_1/2_ shift back to higher binding energies of 711.6 eV and 458.7 eV, respectively (ΔBE of +0.1 eV and +0.3 eV relative to pristine Fe_2_TiO_5_), indicating electron consumption by surface adsorbed species (e.g., CO_2_ or H_2_O) or recombination with holes, while C 1*s* and N 1*s* shift back to lower binding energies of 284.6 eV and 399.8 eV, respectively, suggesting partial re-reduction of the oxidized PDA or the establishment of a new equilibrium under sustained illumination.

These opposite shifts between the metal centers (Fe and Ti) and the organic component (C and N) provide direct spectroscopic evidence for the S-scheme charge transfer mechanism. Under light excitation, photogenerated electrons migrate from PDA to Fe_2_TiO_5_, while holes migrate in the opposite direction [42,43]. The electrons accumulated in the conduction band of Fe_2_TiO_5_ are subsequently trapped by CO_2_ molecules pre-concentrated on the catalyst surface. The abundant amine groups in PDA significantly enhance CO_2_ adsorption, bringing CO_2_ into proximity with the active sites. The adsorbed CO_2_ molecules accept electrons and undergo reduction to CO, with protons supplied by the oxidation of H_2_O. The synergistic combination of enhanced visible light absorption, efficient charge separation, and improved CO_2_ adsorption accounts for the superior performance of the Fe_2_TiO_5_-PDA heterojunction.

## 3. Teaching Suggestions

Through teaching practice with third-year environmental engineering students, the virtual simulation pre-training significantly reduced operational errors in lab experiments, improved success rates, and deepened students’ understanding of advanced characterization techniques such as XPS and CO_2_-BET. Most students were able to establish a clear link between material structure, photophysical behavior, catalytic performance, and reaction mechanism. Future improvements include developing molecular dynamics simulations for PDA polymerization on Fe_2_TiO_5_, building a shared spectral database for student-led comparative analysis, integrating the module into relevant courses, and encouraging peer review and innovative material design proposals. More specifically, the teaching process follows a three-stage route. In the pre-class stage, students’ complete virtual safety training, instrument familiarization, principal animation, and a short online quiz. In the in-class stage, each four-student group performs real synthesis and selected tests, while comparing their own data with virtual standard data to identify possible operational deviations. In the post-class stage, students submit a research-style report that explains the structure-performance-mechanism relationship and proposes one improvement to the catalyst or to the teaching platform.

## 4. Materials and Methods

### 4.1. Synthesis of Catalysts

#### 4.1.1. Synthesis of PDA

A certain amount of DA·HCl (e.g., 5.0 mg) was ultrasonically dispersed in a mixed solvent comprising 30 mL of ethanol and 70 mL of deionized water. Then, 0.6 mL of aqueous ammonia was added, and the mixture was reacted in a water bath at 30 °C for 24 h under magnetic stirring. The resulting solid was collected by centrifugation, washed three times with deionized water and three times with ethanol, and finally freeze-dried to obtain pure PDA.

#### 4.1.2. Synthesis of Fe_2_TiO_5_

In total, 1.5 g PVP was first dissolved in a mixture of 5 mL of DMF and 5 mL of ethanol. The solution was then magnetically stirred for 120 min at room temperature to form a viscous precursor solution. Then, 0.45 mL of butyl titanate and 1.212 g of Fe(NO_3_)_3_·9H_2_O were added to the precursor solution and stirred for 12 h. Subsequently, the prepared solution was put in a 10 mL plastic syringe equipped with a stainless-steel nozzle. A high-voltage power supply of 20 kV was applied at a distance of 15 cm and a flow rate of 0.6 mL/h. After the electrospinning process, the fibers were collected and heat-treated at 250 °C for 1 h, which were calcined at 650 °C for 4 h and then naturally cooled down to room temperature.

#### 4.1.3. FTOP

In a typical procedure, 30 mg of the as-prepared Fe_2_TiO_5_ powder was ultrasonically dispersed in a mixed solvent containing 30 mL of ethanol and 70 mL of deionized water. A specific amount of dopamine hydrochloride (DA·HCl) was added to the dispersion: 1.0 mg for FTOP-1, 2.0 mg for FTOP-2, and 3.0 mg for FTOP-3. The mixture was magnetically stirred to ensure uniform mixing. Subsequently, 0.6 mL of aqueous ammonia (NH_3_·H_2_O) was introduced, and the reaction mixture was placed in a water bath at 30 °C for 24 h under continuous stirring. After the reaction, the solid product was collected by centrifugation, washed three times with deionized water and three times with ethanol, and finally freeze-dried. The obtained samples were denoted as Fe_2_TiO_5_-PDA-1.0 (FTOP-1), Fe_2_TiO_5_-PDA-2.0 (FTOP-2), and Fe_2_TiO_5_-PDA-3.0 (FTOP-3) according to the amount of DA·HCl used. Thermogravimetric (TG) curve of FTOP-2 measured under an air atmosphere. A weight loss of ~1% is observed between 300 and 600 °C, corresponding to the thermal oxidative decomposition of the PDA coating (Figure S3). Based on our previous work [35], pristine Fe_2_TiO_5_ shows no weight loss over 100–800 °C, confirming that the mass change originates exclusively from PDA. The actual PDA loading in FTOP-2 is thus determined to be approximately 1 wt%.

### 4.2. Teaching Methods

The experimental teaching adopts the “virtual simulation pre-training + offline practical verification” hybrid mode (12 credit hours in total), which is consistent with the teaching reform of Fe-based nanomaterial synthesis experiments: (i) A 2-credit-hour virtual simulation for safe practice of autoclave operation, instrument loading (XRD, SEM, in situ XPS), photocatalytic CO_2_ system setup, and GC analysis. (ii) An 8-credit-hour in-class session where students (4 per group) rapidly synthesized Fe_2_TiO_5_-PDA; performed basic characterizations and photocatalytic tests; and jointly analyzed both virtual and measured XRD, SEM, and in situ XPS data. (iii) A 2-credit-hour post-class stage for writing a short research paper on the heterojunction’s effect on charge carrier separation, followed by online discussions. The design is mapped to explicit learning objectives: after the virtual stage, students should be able to describe the safety risks and operating sequence of the experiment; after the offline stage, they should be able to synthesize the catalyst and obtain reliable performance data; after the post-class stage, they should be able to explain the S-scheme mechanism using multiple characterization results. The final score is recommended to include virtual pre-training completion (20%), offline operation and safety performance (30%), data analysis and mechanism explanation (30%), and research-report quality and innovation proposal (20%).

## 5. Conclusions

In summary, this work not only develops a high-performance Fe_2_TiO_5_-PDA S-scheme heterojunction photocatalyst for CO_2_ reduction but also constructs a virtual–real hybrid experimental teaching mode for inorganic chemistry. This mode solves the teaching difficulties of high-risk and high-cost photocatalytic experiments, standardizes students’ operational skills, and realizes the integration of scientific research and teaching. The heterojunction exhibits a CO evolution rate of 14.1 μmol·g^−1^·h^−1^, 6.6 times higher than that of pristine Fe_2_TiO_5_, with 94.2% activity retained after five cycles. Comprehensive characterizations (XRD, SEM, DRS, PL, CO_2_-BET, and in situ XPS) reveal that the enhanced performance stems from efficient charge separation, improved CO_2_ adsorption, and maintained strong redox potential via the S-scheme charge transfer pathway. Teaching practice demonstrates that the virtual–actual hybrid approach effectively reduces operational errors, deepens understanding of advanced instrumentation, and fosters systematic research thinking. From the perspective of experimental teaching reform, the main contribution is the conversion of a frontier CO_2_ reduction study into a structured undergraduate learning module. The proposed route can be extended to other catalyst systems, environmental remediation experiments, and instrumental-analysis courses that require students to integrate synthesis, characterization, performance evaluation, and mechanism discussion.

## Data Availability

Data are contained within the article and Supplementary Materials.

## Supplementary Materials

The following supporting information can be downloaded at https://www.mdpi.com/article/10.3390/molecules31101703/s1: Text S1: Characterization. Text S2: Photocatalytic CO_2_ reduction experiments. Figure S1: SEM images of Fe_2_TiO_5_ at different resolutions: (a) 10 μm and (b) 5 μm. Figure S2: EDS mapping of carbon element in FTOP-2. Figure S3: TG curve of FTOP-2. Figure S4: Results of control experiments for photocatalytic CO_2_ reduction. Figure S5: GC-MS spectra of the products from the ^13^CO_2_ isotopic labeling experiment over FTOP-2 under visible light irradiation. Figure S6: Tauc curve of Fe_2_TiO_5_. Table S1: Comparison of the performance of photocatalytic CO_2_ reduction to produce CO Refs [44,45,46,47,48,49,50,51]. Table S2: Binding energies and chemical state assignments in Fe_2_TiO_5_, PDA, and FTOP-2 under dark and light illumination conditions.
