# Supplementary material for: Design of Fe2TiO5-PDA Heterojunction for Photocatalytic CO2 Reduction: From Mechanism Research to Virtual–Real Hybrid Chemistry Experimental Teaching Reform
Source: Molecules. 2026 May 18;31(10):1703. doi: 10.3390/molecules31101703 (PMC13209323; doi:10.3390/molecules31101703)
Supplement: Supplementary file 1 [file molecules-31-01703-s001.zip › molecules-4302962-supplementary.pdf]

Design of Fe_2TiO_5-PDA heterojunction for photocatalytic CO_2 reduction: From mechanism research to virtual-real hybrid chemistry experimental teaching reform

Kai Wang[1,3]*, Yihui Du[1] and Liang Wang[2]*

[1] College of Urban and Environmental Sciences, Hubei Normal University, Huangshi, 435002, P. R. China

[2] Institute of Nanochemistry and Nanobiology, School of Environmental and Chemical Engineering, Shanghai University, Shanghai, 200444, P. R. China

[3] School of Chemistry, Chemical Engineering and Biotechnology, Nanyang Technological University, Singa-pore 637459, Singapore

* Correspondence: wangkai@hbnu.edu.cn; wangl@shu.edu.cn

Text S1 Characterization

X-ray diffraction (XRD) measurements were performed using Cu K-α radiation from a Rigaku Co. D/max RA model. The morphological and structural characteristics of the photocatalyst were examined with a transmission electron microscope (TEM) model X-MaxN20 (JSM-IT300) and high-angle annular dark-field scanning transmission electron microscopy (HAADF-STEM, Titan G2 60-300). In situ XPS was performed on a VG ESCALAB250 surface measurement system with an Al Kα X-ray source (hv = 1486.6 eV, beam spot: 400 μm) under a vacuum better than 5.0×10^{-7} mbar. For the light-on condition, the sample was irradiated with a 375 nm UV light source (power > 15 mW) for 15 minutes prior to and during data acquisition, while the dark condition spectra were collected without irradiation. UV-Vis diffuse reflectance spectra (UV-Vis DRS) were collected over a wavelength range of 300 to 2000 nm using a Shimadzu UV-2550 spectrophotometer. CO_2 adsorption isotherms were measured on a Micromeritics ASAP 2460 analyzer at 25 °C (298 K). Prior to measurement, samples were degassed under vacuum at 120 °C for 6 h. The adsorption data were collected over an absolute pressure range of approximately 385–761 mmHg (~0.51–1.01 bar) with an equilibration interval of 15 s. The adsorption uptake is reported in $cm^3 \cdot g^{-1}$ (STP), normalized per gram of sample mass.

Text S2 Photocatalytic CO_2 reduction experiments

The photocatalytic reduction of CO_2 with H_2O was conducted in a custom-designed sealed reaction vessel. Initially, 30 mg of the photocatalyst was dispersed in deionized water through ultrasonication and placed into a crucible cover. After drying, the crucible cover was positioned in the reactor, and 2 mL of deionized water was added to the bottom of the reactor to seal the reaction chamber. The vessel was then evacuated with a vacuum pump to eliminate any ambient gases. Following this, high-purity CO_2 was introduced into the evacuated reactor. A 300 W Xe lamp (PLS-SXE300D, Perfectlight) equipped with a UV cut-off filter ($\lambda > 420$ nm) was used as the visible light source. The light intensity at the sample position was approximately 200 $mW \cdot cm^{-2}$, with a lamp-to-reactor distance of ~10 cm and a spot diameter of ~5 cm. The gaseous products were analyzed by gas chromatography using a flame ionization detector (FID) for CO and CH_4, with calibration performed using standard gas mixtures.

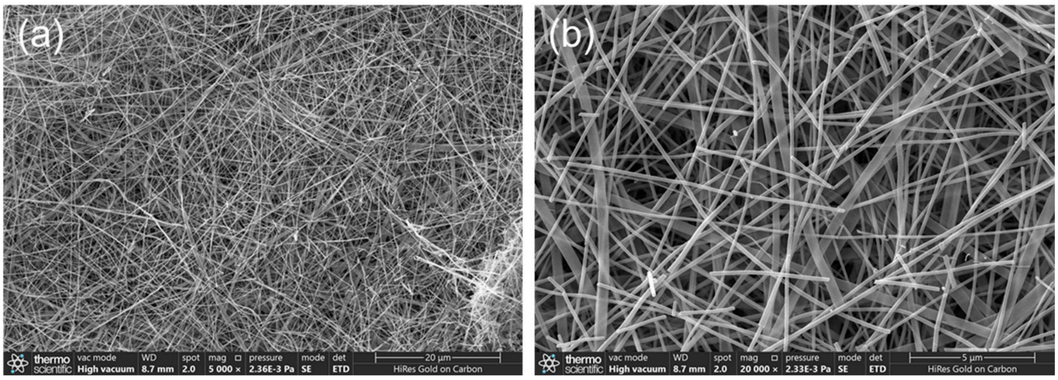

Figure S1 SEM images of Fe_2TiO_5 at different resolutions: (a) 10 μm and (b) 5 μm.

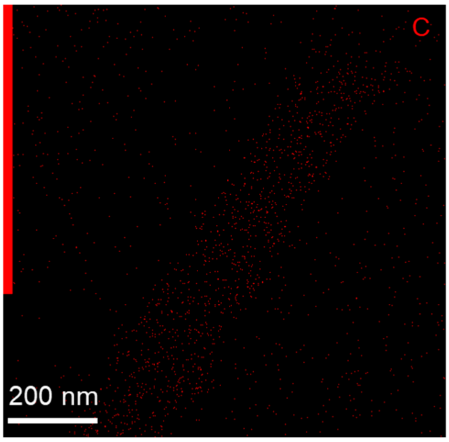

Figure S2 EDS mapping of carbon element in FTOP-2

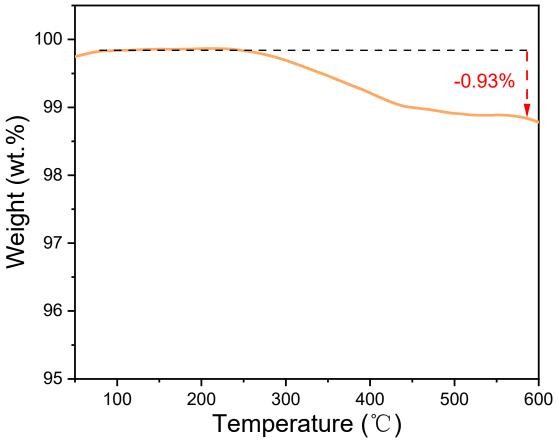

Figure S3 TG curve of FTOP-2.

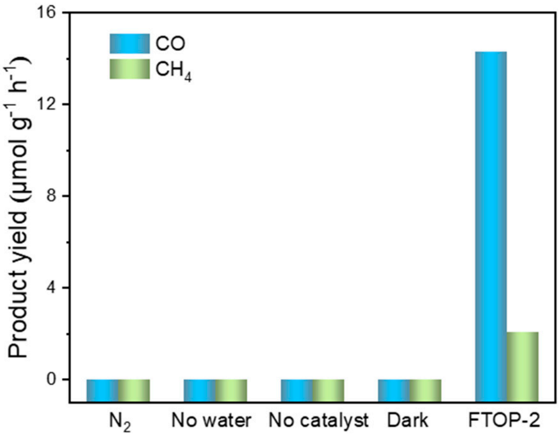

Figure S4 Results of control experiments for photocatalytic CO_2 reduction.

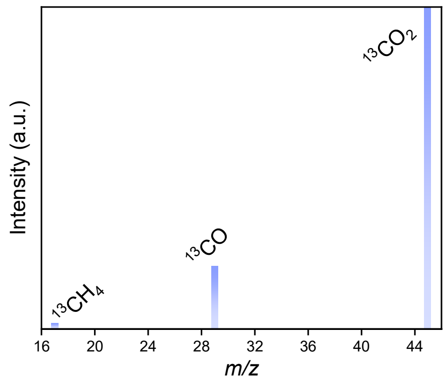

Figure S5 GC-MS spectra of the products from the $^{13}CO_2$ isotopic labeling experiment over FTOP-2 under visible light irradiation.

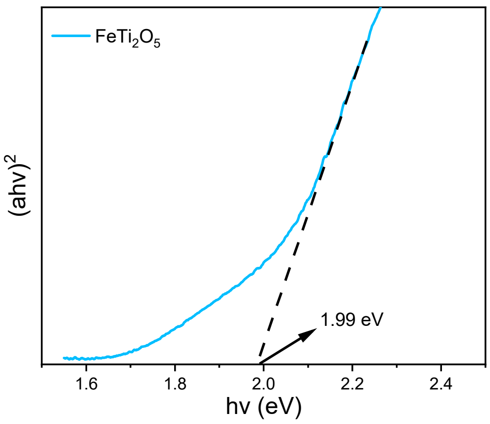

Figure S6 Tauc curve of Fe_2TiO_5.

Table S1 Comparison of the performance of photocatalytic CO_2 reduction to produce CO.

Catalyst	Light source	CO production rate (μmol $g^{-1} h^{-1}$)	Reference
11CN	300 W Xe lamp	2.13	[1]
Ag/TiO_2	300 W Xe lamp	0.575	[2]
Cu/Cu_2O	300 W Xe lamp	8.5	[3]
g-C_3N_4/Bi/carbon dots	300 W Xe lamp	9.08	[4]
MoO_{3-X}-TiO_2	Visible light	12.0	[5]
$Bi_2WO_6/Cd_{0.3}Zn_{0.7}S$	Visible light	12.02	[6]
GaO/LaOCO	300 W Xe lamp	3.06	[7]
$Cs_3Bi_2I_9/In_2S_3$	300 W Xe lamp	10.4	[8]
FTOP-2	Visible light	14.1	This work

Table S2. Binding energies and chemical state assignments in Fe_2TiO_5, PDA, and FTOP-2 under dark and light illumination conditions.

Element	Sample	Chemical State	Binding Energy (eV)	ΔBE vs. Fe_2TiO_5/PDA (eV)
	Fe_2TiO_5	Fe^{3+}	711.5	—
Fe $2p_{3/2}$	FTOP-2 dark	Fe^{3+}	710.8	−0.7
	FTOP-2 light	Fe^{3+}	711.6	+0.1
	Fe_2TiO_5	Ti^{4+}	458.4	—
Ti $2p_{1/2}$	FTOP-2 dark	Ti^{4+}	458.3	−0.1
	FTOP-2 light	Ti^{4+}	458.7	+0.3
	PDA	C–C (aromatic)	284.8	—
C 1s	FTOP-2 dark	C–C (aromatic)	285.0	+0.2
	FTOP-2 light	C–C (aromatic)	284.6	−0.2
	PDA	C–N / pyrrolic N	399.8	—
N 1s	FTOP-2 dark	C–N / pyrrolic N	400.2	+0.4
	FTOP-2 light	C–N / pyrrolic N	399.8	0

References

1. Hu, D.; Li, S.; Chen, Y.; Shen, Y.; Liao, X.; Zhou, X.; Xu, H.; Tan, Y.; Zhong, J. Nitrogen vacancies enriched g-C_3N_4 nanosheets for photocatalytic CO2 reduction. *Int. J. Hydrogen Energy* **2025**, 158, 150571.
2. Li, G.; Sun, Y.; Zhang, Q.; Gao, Z.; Sun, W.; Zhou, X. Ag quantum dots modified hierarchically porous and defective TiO_2 nanoparticles for improved photocatalytic CO_2 reduction. *Chem. Eng. J.* **2021**, 410, 128397.
3. Sayed, M.; Zhang, L.; Yu, J. Plasmon-induced interfacial charge-transfer transition prompts enhanced CO_2 photoreduction over Cu/Cu_2O octahedrons. *Chem. Eng. J.* **2020**, 397, 125390.
4. Zhao, X.; Li, J.; Kong, X.; Li, C.; Lin, B.; Dong, F.; Yang, G.; Shao, G.; Xue, C. Carbon dots mediated in situ confined growth of Bi clusters on g- C3N4 nanomeshes for boosting plasma-assisted photoreduction of CO_2. *Small* **2022**, 18, e2204154.
5. Xie, S.; Zhang, H.; Liu, G.; Wu, X.; Lin, J.; Zhang, Q.; Wang, Y. Tunable localized surface plasmon resonances in MoO_{3-x}-TiO_2 nanocomposites with enhanced catalytic activity for CO_2 photoreduction under visible light. *Chin. J. Catal.* **2020**, 41, 1125-1131.
6. Niu, L., Liu, X., Liu, X., Yu, J., Chen, R., Yang, K., Chen, L., Hu, M., Wu, J., Hosseini-Bandegharaei, A. Constructing a 3D Bi_2WO_6/$Cd_{0.3}Zn_{0.7}S$ heterojunction to enhance photocatalytic CO_2 reduction efficiency. *J. Environ. Chem. Eng.* **2026**, 14, 121167.
7. Wang, X., Liu, Y., Xu, A., Zhang, Q., Yang, G., Tong, L., Peng, F. CO_2 mass transfer optimization for enhanced photocatalytic CO_2 reduction via a surface CO_3^{2-} layer reconstruction. *Surf. Interfaces* **2026**, 88, 109097
8. Li, N., Liao, Y.-T., Wang, K., Wang, S.-Y., Zhang, Y.-F., Ma, Y.-L., Zhong, M.-L., Xiao, M.-J., Li, Y.-Y., Sun, W.-J., Wang, Q. Rational design an S-scheme heterostructure $Cs_3Bi_2I_9$/In_2S_3 derived from In-MOF for boosted photocatalytic CO_2 reduction. *J. Alloys Compd.* **2026**, 1061, 187513.
